# Eavesdropping on Tinnitus Using MEG: Lessons Learned and Future Perspectives

**DOI:** 10.1007/s10162-023-00916-z

**Published:** 2023-11-28

**Authors:** Lisa Reisinger, Gianpaolo Demarchi, Nathan Weisz

**Affiliations:** 1 Centre for Cognitive Neuroscience and Department of Psychology, Paris-Lodron-University Salzburg, Salzburg, Austria; 2 Neuroscience Institute, Christian Doppler University Hospital, Paracelsus Medical University, Salzburg, Austria

**Keywords:** Tinnitus, Magnetoencephalography, Review, Resting state, Tone stimulation

## Abstract

Tinnitus has been widely investigated in order to draw conclusions about the underlying causes and altered neural activity in various brain regions. Existing studies have based their work on different tinnitus frameworks, ranging from a more local perspective on the auditory cortex to the inclusion of broader networks and various approaches towards tinnitus perception and distress. Magnetoencephalography (MEG) provides a powerful tool for efficiently investigating tinnitus and aberrant neural activity both spatially and temporally. However, results are inconclusive, and studies are rarely mapped to theoretical frameworks. The purpose of this review was to firstly introduce MEG to interested researchers and secondly provide a synopsis of the current state. We divided recent tinnitus research in MEG into study designs using resting state measurements and studies implementing tone stimulation paradigms. The studies were categorized based on their theoretical foundation, and we outlined shortcomings as well as inconsistencies within the different approaches. Finally, we provided future perspectives on how to benefit more efficiently from the enormous potential of MEG. We suggested novel approaches from a theoretical, conceptual, and methodological point of view to allow future research to obtain a more comprehensive understanding of tinnitus and its underlying processes.

**Supplementary Information:**

The online version contains supplementary material available at 10.1007/s10162-023-00916-z.

## Introduction

Tinnitus is a phenomenon experienced by 10–15% of the population, with its prevalence increasing to 24% in individuals beyond 65 years [1, 2]. Approximately 1–3% of the population experience bothersome tinnitus, which significantly affects their quality of life due to associated symptoms such as reduced sleep quality, increased distress, or anxiety [3, 4]. Despite these numbers, our current understanding of the underlying mechanisms is unsatisfactory, hindering the development of effective treatments. Any scientific theory aiming to “explain” tinnitus must consider a few essential facts: (1) The vast majority of individuals with tinnitus simultaneously experience at least some mild form of hearing loss [5, 6]. Thus, by a wide margin, hearing damage is the most important risk factor. (2) Tinnitus is not solely linked to hearing damage but is often also characterized by its heterogeneity and comorbidity. Depending on the severity of the tinnitus distress, tinnitus patients suffer more from general psychopathology such as depressive symptoms and show stronger somatization [7]. Moreover, cognitive impairments were reported which, among others, can affect speech comprehension [8]. These additional factors are widely ignored; however, they can restrict controlled tinnitus research. (3) Intuitively, one could assume that tinnitus results from, e.g., sounds generated by the inner ear or increased firing of hearing nerve fibers [9, 10]. However, while the initiating event may be of peripheral origin, the persistence of tinnitus is unlikely to be maintained by the inner ear (see data on resection of hearing nerve [11, 12]). (4) Not all individuals with hearing loss experience tinnitus [6]. (5) Not all individuals with tinnitus are distressed to the same extent [13]. In combination, these facts have driven an increasing number of scientists to seek answers in the brain. This involves not only identifying the source of neural activity responsible for sound perception (the “neural correlate,” using a somewhat overused term) but also explaining the considerable interindividual variability observed in tinnitus. Notably, comparing neural activity of individuals with and without tinnitus is further characterized by the ubiquitous presence of sound perception in the tinnitus group. Therefore, a tinnitus “resting” brain (i.e., without resolving any active tasks) differs from a healthy control since neural activity in the tinnitus group always reflects the perception of a sound. While some neuroscience efforts have employed animal models following experimental manipulations to induce tinnitus-like conditions, studies in humans have predominantly focused on comparing groups of individuals with or without tinnitus or exploring different “subtypes” of tinnitus, such as high vs low distress [14–16]. In human studies, noninvasive approaches are typically employed, with electroencephalography (EEG) being the primary method due to its cost-effectiveness and widespread availability. However, magnetoencephalography (MEG) has also been utilized to gain a deeper understanding of tinnitus [14]. The purpose of this review is manifold: Firstly, we aim to introduce this tool to interested tinnitus researchers. Secondly, a (in some parts critical) synopsis of the current state will be provided, outlining inconsistencies and limitations. Finally, to end on a more positive note, we want to sketch future perspectives and guidelines on how to better exploit the enormous potential of MEG. To realize these goals, we need to start with some basic ideas, on how tinnitus could be generated by neural activity, which will then lead us to why and in what form this activity could possibly be picked up non-invasively.

## Concepts Linking Tinnitus to Neural Activity

**Fig. 1:**
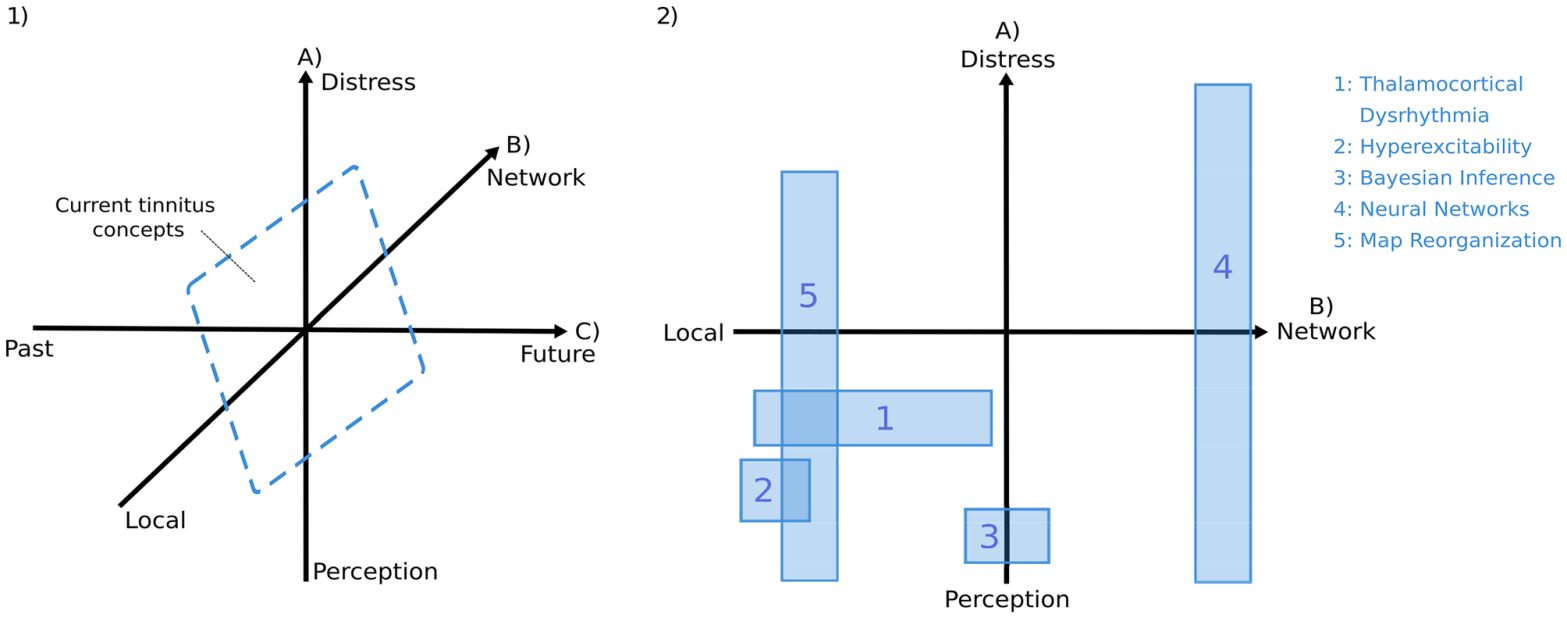
Dimensions of current tinnitus theoretical concepts to approach the generation and preservation of tinnitus. (1) Integration of tinnitus concepts along the three dimensions (A) perception-distress, (B) local-network, and (C) past-future. Dimension (A) refers to the different focus of various tinnitus frameworks either on the perceptual or on the distressing aspect of tinnitus. Dimension (B) highlights the neuroanatomical focus of the frameworks that is either particularly on the auditory system or more widespread on larger brain networks. Dimension (C) comprises the time aspect within the tinnitus frameworks, meaningly the reflections of processes in the past as well as how the current state can predict future trajectories. (2) Relevant tinnitus frameworks guiding past MEG research arranged along the two dimensions (A) perception-distress and (B) local-network

Over the years, various approaches have been developed to explain the generation of tinnitus. Models vary with regard to their scope (i.e., the explanandum) emphasizing to different degrees either the perceptual or the distressing aspect of tinnitus (axis A in Fig. 1). This aspect partially correlates with the neuroanatomical focus of the respective model (axis B in Fig. 1), which ranges from, e.g., activational abnormalities in specific regions of the ascending auditory system (e.g., dorsal cochlear nucleus, primary auditory cortex) to more generalized alterations in larger networks within the brain. Regarding the aspect of time (axis C in Fig. 1), it is crucial to understand both the processes in the past that led to the current tinnitus-state as well as how the current state can predict future trajectories. We will specify some of the most popular theories in more detail, varying from local dysfunctions to more widespread “networks” of brain regions.

Early, neurocentric ideas inspiring MEG research sometimes emphasized the similarities of tinnitus to phantom limb pain [10, 17–19]. As for the latter, deafferentation (here: hearing loss) seems to be at the outset of tinnitus [20], leading to map reorganization in auditory regions [21–24]. These effects of tonotopic reorganization are thought to result from a reduction of neural inhibition [4, 25]. Apart from auditory regions, reorganization includes limbic and thalamic structures as well, and changes are further characterized by hyperactivity and increased synchronous neural activity in the affected areas [20, 26–28]. However, uncertainty remains regarding whether this reorganization promotes [29] or reduces tinnitus [30]. Moreover, in human studies, the presence of map reorganization in tinnitus has not been consistently demonstrated [31]. Research has further suggested that cortical reorganization is more pronounced in cases of hearing loss than in those with additional tinnitus [32, 33]. As a result, findings regarding this approach as the underlying mechanism of tinnitus per se remain indecisive.

Another approach to explain tinnitus has placed more emphasis on features of ongoing (“spontaneous”) neural activity. One of the pioneering frameworks in this regard is the thalamocortical dysrhythmia hypothesis [34], which posits that tinnitus arises as a consequence of altered neural coherence in thalamocortical regions. Crucially, this model was the first to highlight the role of neural oscillations in tinnitus. The core concept revolves around deafferentation-induced changes in firing patterns, leading to slow wave oscillations in thalamic structures and subsequently affecting thalamocortical connections in the corresponding auditory cortical areas. Moreover, the framework suggests that slow-wave activity in specific auditory cortical areas results in enhanced gamma activity in surrounding regions due to reduced lateral inhibition. Notably, a central prediction of this framework is that enhanced gamma activity should underlie the perception of the phantom sound, an idea that has been instrumental in many MEG/EEG-based studies [35–37].

Another popular deafferentation-based approach emphasizes hyperexcitability in auditory processing regions. According to this notion, reduced auditory input due to hearing damage leads to an increased gain in the central auditory pathway [38]. This hyperexcitability could be related to homeostatic plasticity, wherein affected neurons attempt to restore their previous activity level by responding more strongly to any given input. Moreover, this approach predicts increased spontaneous activity in the auditory pathway, ultimately resulting in the perception of tinnitus [39]. Unlike the thalamocortical dysrhythmia hypothesis, this framework does not assign a special role to oscillations, although they are not explicitly excluded either.

A common thread running through all the described frameworks is that they are based on the presence of hearing loss and the resulting altered ongoing activity pattern, which reflects a disturbed excitatory-inhibitory balance. Apart from a lack of conclusive and robust evidence in humans, there are further explanatory gaps [38]. Firstly, most individuals with hearing loss do not develop tinnitus, indicating that auditory deprivation is insufficient in explaining this condition [6]. Further, tinnitus and hearing loss onsets do not often occur at the same time and not all cases of acute tinnitus become chronic [40, 41].

A current approach to overcome these explanatory gaps is the Bayesian inference framework [42]. In this framework, internal models are emphasized to construct perception [43]. The principle of predictions (or so-called priors) is important in this framework as they are constantly compared to sensory input. Information from the environment is therefore used to update prior beliefs to posterior assumptions. Thus, the brain aims to make predictions about upcoming events and updates these predictions in a Bayesian way [44]. Auditory deafferentation is therefore processed as a prediction error as it does not assemble memory-based priors [17]. Spontaneous activity in the auditory pathway acts therein as a precursor of tinnitus. This spontaneous activity is ignored in a healthy system, since the default prior is silence. However, in case these priors are shifted towards the expectation of a sound, phantom perceptions can be perceived [42, 45]. Empirical evidence for this framework is still sparse, since it is difficult to measure hypothetical tinnitus-related priors from ongoing brain activity. The few sound-stimulation based studies however lend some support for the idea that prediction processes in tinnitus are altered [46–49]. However, since findings targeting the theoretical framework of hyperexcitability are inconclusive (see, e.g., [50]), the Bayesian inference framework depicts a powerful novel approach that can accommodate the fact that non-invasive proxies of “enhanced excitability” are not robustly found. Moreover, invasive studies with animals could also not consistently report enhanced excitability in tinnitus [51–53]. Furthermore, it is left unclear when and why “default priors” should change.

While previous frameworks mainly attempt to explain the perception aspect of tinnitus, others also try to cover the variable levels of distress as well as the influence of other psychological factors such as attention. These mostly exceed the level of auditory processing regions to include distributed neural networks (see [54], for a neural network model of tinnitus perception). As a paradigmatic example, De Ridder et al. [17] proposed a model of tinnitus involving multiple networks, including the memory network, distress network, loudness network, and salience network. This suggests that various brain regions and networks are implicated in the experience of tinnitus, and changes in connectivity within attentional and emotional networks might also play a role [55]. Numerous studies have consistently shown modifications in these networks in individuals with tinnitus [56]. In bothersome and chronic tinnitus cases, researchers have found decreased connectivity between the auditory cortex and visual, attention, and control networks [57, 58]. This implies that disruptions in the communication between these networks may contribute to the perception and distress associated with tinnitus. Recently, De Ridder et al. [59] proposed a novel approach that involves a combination of a sound pathway, a suffering pathway, and a noise-canceling pathway to explain tinnitus and its comorbidities in a triple network.

Sedley et al. [42] comprehensively stated that most of these “network”-based models are problematic for several reasons: (1) the concepts propose contradictory alterations in activity or connectivity, (2) it has not been sufficiently demonstrated that spontaneous activity or synchrony is enhanced in tinnitus irrespective of hearing loss, and (3) the models require multiple origins of tinnitus generation. We add to this criticism that, next to the importance of hearing damage [5, 6], neuro-developmental aspects that promote tinnitus development are widely ignored. For example, we have recently shown in independent samples that chronological age increases the risk of tinnitus in addition to hearing loss [60]. This points to the importance of latent biological aging processes that still need to be understood. Overall, current tinnitus frameworks do not take the aspect of time into account seriously (axis C in Fig. 1). Understanding which processes in the past have led to the current tinnitus-state as well as how the current state predicts future trajectories will not only be important from a basic science perspective (axis C in Fig. 1), but also play a critical role when it comes to treatment and, in particular, prevention of tinnitus.

**Fig. 2:**
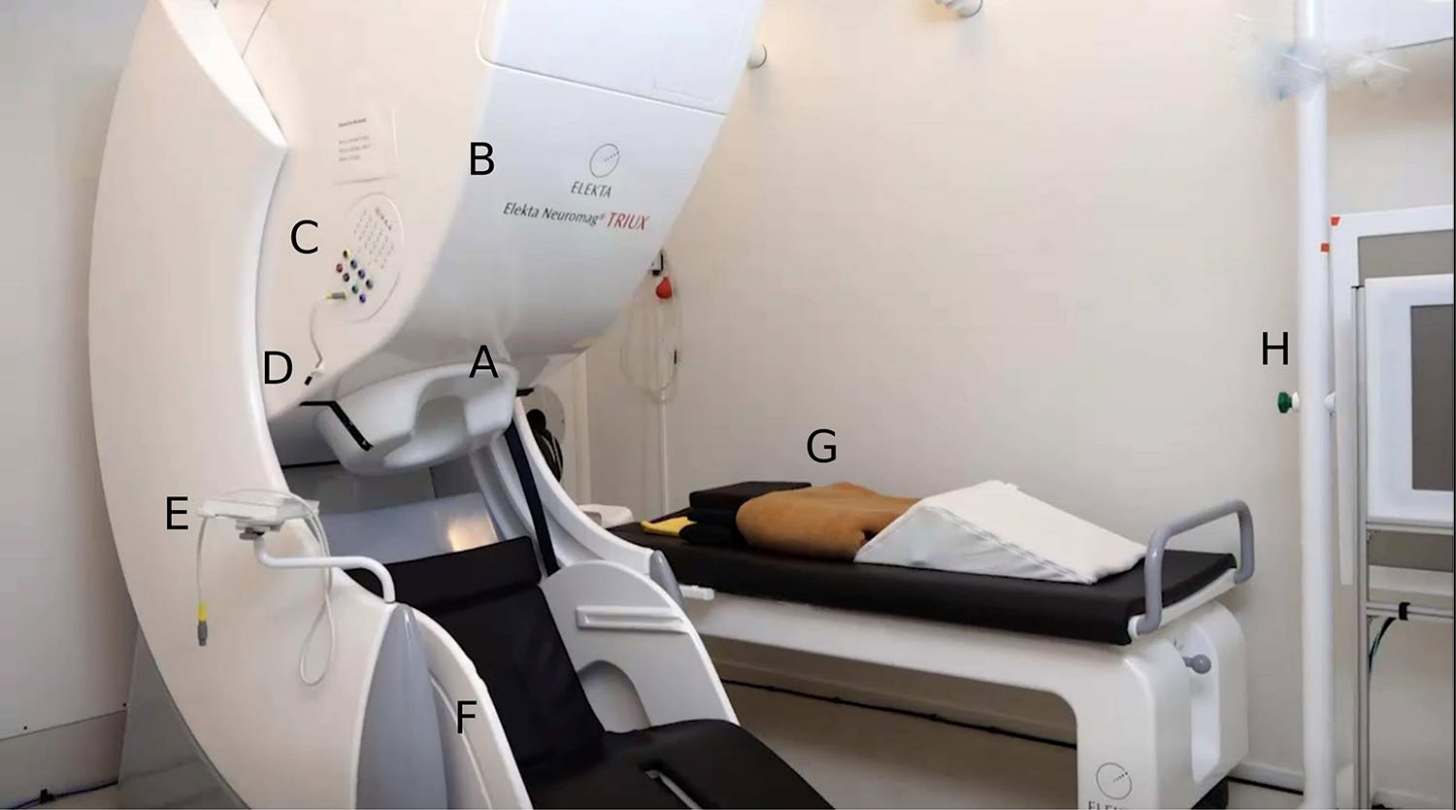
Magnetoencephalography (device: whole-head MEG Triux, MEGIN Oy, Finland) within the magnetically shielded room (AK3b, Vacuumschmelze, Germany). **A** MEG-helmet with SQUID-sensor-unit. **B** Dewar with liquid helium inside. **C** EEG-unit with inputs for electrical signals (e.g., EOG, ECG). **D** Microphone. **E** Inputs for single EEG-electrodes. **F** MEG seat. **G** MEG bed. **H** Screen for visual stimulation

Box 1 Magnetoencephalography Magnetoencephalography (MEG) is a state-of-the-art technique used to measure the magnetic fields generated by neuronal activity in the brain (see Fig. 2). David Cohen first introduced the method in the 1960s, utilizing a single resistive detector (magnetometer) to measure human magnetic brain activity [61]. With the subsequent advent of superconducting sensors (SQUIDs), MEG has evolved into a sophisticated technology for non-invasive measurement with high temporal and spatial resolution of the underlying neuronal activity using sensors located on the scalp [62–64]. The synchronous activations of 10,000 to 50,000 neurons produce magnetic fields strong enough to measure from the outside the head [65]. One of the major advantages of MEG over other neurophysiological techniques, such as electroencephalography (EEG), is that the magnetic fields generated by the brain pass through the head tissues essentially undistorted, resulting in more focal activity and potential better source localization [66, 67]. In terms of spatial resolution, MEG measurements are worse than blood-flow imaging techniques like fMRI but still highly accurate. Research has found evidence for sufficient MEG recordings even in deep-brain structures like amygdala, hippocampus, or thalamus [68, 69]. However, the measurement of magnetic fields from the brain poses some challenges due to the extremely weak signals compared to external noise [70, 71]. To obtain measurements of sufficient quality, a magnetically shielded room is required to reduce the interference caused by external sources, including electrical equipment, motors, and others. Additionally, metallic objects in the participant’s body such as dental braces, cochlear implants, or hearing aids can further disturb the magnetic brain fields, affecting the accuracy of the results [66, 72]. Due to these circumstances, auditory stimulation in the MEG cannot reach excellent sound quality since in most cases sounds have to be delivered through pneumatic tubes from outside the shielded room. Despite these limitations, MEG has proven to be a valuable tool for studying various cognitive functions and has been used in numerous research studies in areas such as language processing, visual and somatosensory perception, motor control, and more. The technique has also been used in clinical settings to diagnose and monitor neurological disorders such as epilepsy and to evaluate the effectiveness of treatments.

MEG is particularly useful for auditory research, as the shielded room allows recordings to be made in a silent environment compared to the noisy setting in MRI measurements [14]. Additionally, the time to prepare for measurements is shorter compared to EEG studies which is especially suitable for investigating patients. As a conclusion, with the high temporal resolution, whole-head coverage of more than 300 sensors, and good spatial resolution the MEG represents an outstanding tool to investigate tinnitus mechanisms [73]. The technique has also been successfully used to investigate the neural mechanisms of speech and music perception, as well as to identify auditory processing deficits in patients with hearing impairments.

## Why Could We in Principle Measure (Features of) Tinnitus “from the Outside” Using Neuroimaging Techniques?

In general, most tinnitus frameworks make a plausible assumption that conscious perceptions, such as tinnitus, involve a substantial amount of coordinated neural activity within and beyond the auditory system. In such cases, brain activity can potentially cause changes in the electric or magnetic field, which can be captured non-invasively outside the head using appropriate technologies. For this purpose, EEG and MEG are powerful and established tools. While both methods share common underlying biophysical principles in signal generation, MEG often provides superior insights into brain function for several reasons (see Box 1).

**Fig. 3:**
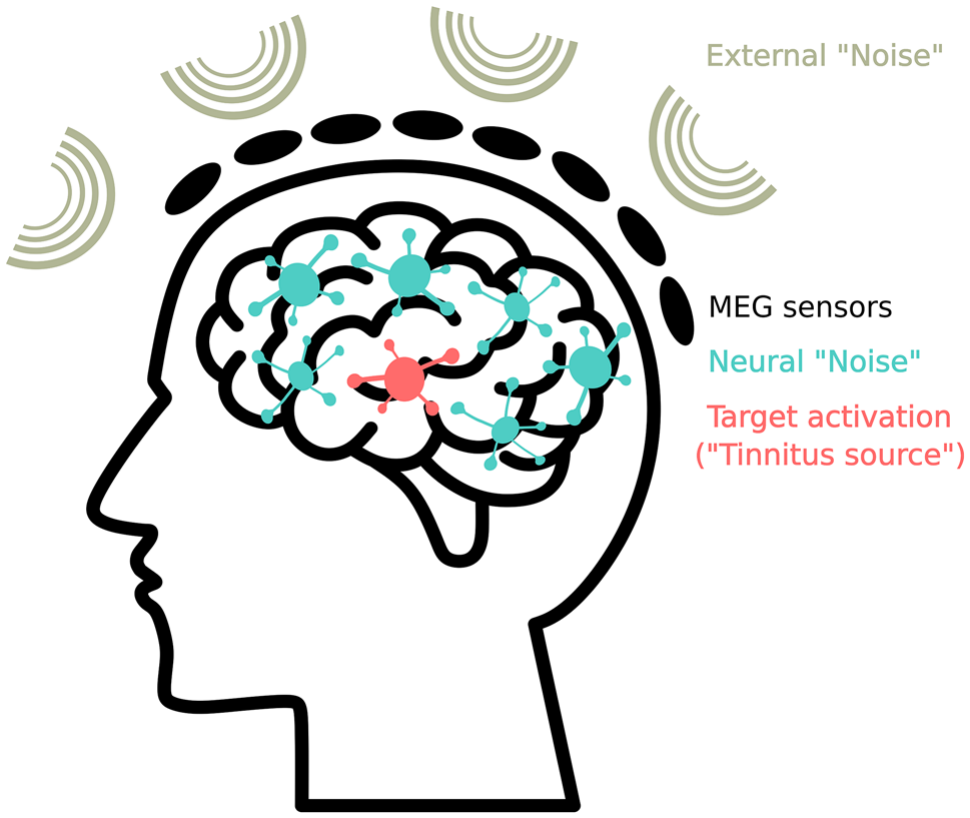
Signal recording of the MEG sensors. We aim to capture the target activation (e.g., some aberrant neural activity as the source of the tinnitus) within the brain using MEG sensors on the scalp. However, the identification of this target activation may be obscured by multiple factors. These include not only genuine external noise, such as environmental disturbances, but also internal activity from other brain regions that are not directly relevant to the investigation of tinnitus

MEG has clear advantages, particularly in the unmixing of signal generators, which is crucial for distinguishing various sources within the brain and separating brain activity from non-neural noise sources as well as neural activity in various brain regions that is not related to tinnitus (Fig. 3) [64, 66]. A detailed explanation of state-of-the-art source analysis approaches goes beyond the scope of this review, but interested readers are encouraged to consult [64, 74–76].

As described previously, most neuroscientific tinnitus frameworks propose some deprivation-related aberrant neural activity to underlie the tinnitus sensation. While most animal models can resort to the “ground truth” of action potentials, it is not trivial how, e.g., “hyperexcitability” is manifested in non-invasive signal. The latter is thought to reflect the temporally synchronized summed excitatory postsynaptic potential of many apical dendritic pyramidal cells [77]. Before delving into specific findings in the MEG literature, it is thus useful to spell out the three main flavors of brain activity studied in general [78, 79] that can be differentiated according to the extent they are (a) exogenously (i.e., stimulus-) driven vs endogenously generated and (b) in stimulus-driven cases, how time- and phase-locked the responses are. Endogenous activity in absence of any external stimulation makes up the category of spontaneous or ongoing brain activity studies, which have taken an outstandingly important position in the human M/EEG tinnitus literature. Stimulus-driven activity comes in two main categories: evoked and induced responses.

Spontaneous activity reflects the fact that the brain is active even without external input and describes ongoing background activity while no specific task is being performed. Different frequencies of oscillations—often summarized in canonical frequency bands—have been attributed to different brain regions as well as cognitive functions (for a review, see, e.g., [77]). Oscillations in various frequency ranges have been used to investigate tinnitus and have been integral to some of the conceptual frameworks, e.g., in the thalamocortical dysrhythmia hypothesis [34] or network models [17, 54]. Spontaneous activity can be analyzed across frequencies in terms of power and amplitude and therefore reveal neural differences in group comparisons between participants with and without tinnitus [14]. Going beyond this “local” perspective of frequency-band restricted neural activity, many resting-state studies are also extended by quantifying the inter-relationships of activity at different recording sites [80], with the goal of providing insights into the network states in tinnitus as compared to control individuals (see, e.g., [55, 57]).

Evoked responses normally result from external stimuli that exhibit neural responses of a certain polarity after a certain onset latency (i.e., phase- and time-locked; in principle, evoked activity could also refer to some internal event such as heartbeats or movements). These neural activations are often analyzed to test ideas on reorganization processes or hyperexcitability in tinnitus, which are revealed by altered responses to stimuli. Phase-locking refers to the synchronization of the phases of oscillatory neural activities at specific frequencies, across trials, and/or sensors. The basic analysis steps for evoked responses are averaging over trials in order to increase signal-to-noise ratio and detect the neural response after a stimulus. The amplitude of the response reflects therein the strength of the neural potentials [78, 81].

Induced responses also require an eliciting event which classically is an external stimulus. However, in contrast to evoked responses, they are not strictly phase-locked and—within certain limits—show variable timing between trials. Consequently, averaging of time series will remove the effect, and spectral analysis methods must be applied to the single trials before averaging [82]. Faster frequency oscillations are assumed to be generated by fewer synchronized neurons, whereas slow oscillations emerge from larger cortical areas of synchronized activity [83]. Therefore, the size of the activated area plays a role in the corresponding frequency and amplitude of the induced response [84].

The differences between spontaneous, evoked, and induced responses clearly show the importance of study design (resting-state vs tone stimulation) and analysis methods to specifically target a certain type of response (Box 2).

Box 2 Common MEG Analysis Approaches in Tinnitus Research In general, MEG methods can be divided in sensor space and source space analyses. Sensor space analyses involve analyzing the signals recorded from each MEG sensor separately, and then using statistical methods to identify patterns in the data that are related to neural activity [85]. Most prominently, evoked response analyses are used to detect stimulus-driven activity in the brain. In terms of auditory brain functioning research and tinnitus, the N1 component of auditory evoked responses, known as N1m in MEG, has been extensively investigated [14]. The N1/N1m depicts a negative wave that peaks around 100 ms after stimulus onset and reflects stimulus properties such as frequency, sound intensity, and amplitude [86, 87]. Moreover, the N1/N1m component is sensitive to auditory selective attention, which is assumed to be relevant in tinnitus as well [86, 88].

Next to the N1m component, auditory steady-state responses (ASSR) were as well reported in many MEG studies analyzing tinnitus. In general, the ASSR is an auditory evoked potential that is used to investigate hearing sensitivity and hearing loss characteristics [89]. Steady-state responses are defined as an evoked response that appears after modulating stimulus presentation rates. If the interstimulus interval is short enough, the response to the previous stimulus has not vanished before the following stimulus is presented [90]. In the auditory dimension, periodically presented acoustic stimuli with a repetition rate between 35 and 40 Hz are supposed to elicit steady-state responses [91]. The ASSR has been located mainly to the primary auditory cortex, exhibiting a tonotopic organization, where lower frequencies are localized more laterally and higher frequencies more medially [92, 93].

Source space analyses aim to derive information from the neural activity modeled at specific locations in the brain. Hence, while sensor space analyses focus on measuring of magnetic fields outside of the head, source space analyses attempt to estimate the location and strength of the sources generating those fields within the brain [85, 94]. Given the massive volume conduction issue, connectivity analysis at the sensor level is not ideal. Source modeling is, therefore, highly relevant in the context of connectivity analyses. The rationale of connectivity analyses is that brain regions should not only be investigated separately but also in relation to other areas that are simultaneously activated and interact with each other [75, 95, 96]. These interacting brain regions further yield a neural network with widespread distribution and specific functionality [97]. This analysis approach is further valuable in tinnitus research to reveal relevant networks in tinnitus generation and perception.

## Findings in MEG

Studies in MEG targeting tinnitus usually follow two design approaches. A large proportion of studies aim to derive insights regarding tinnitus based on resting state measurements. In this case, participants with tinnitus undergo MEG measurements without any stimulation, and solely untargeted spontaneous brain activity is recorded. Usually, the resulting patterns are compared to those of a tinnitus-free control group. An alternative, albeit less commonly practiced approach, is to compare resting state activity before and after some tinnitus-modifying interventions, such as residual inhibition (e.g., [98, 99]) or neurostimulation (e.g., [100]). Another common research direction involves probing neural response patterns to sounds. In this case, participants listen to, e.g., tones with frequencies corresponding similar or different to that of the tinnitus percept. We will address current findings in MEG research separately for these two design approaches.

### Resting State Measurements

First, we will present a compilation of recent tinnitus discoveries that used MEG over the past 10 years, primarily focusing on utilizing resting state measurements. We will approach these findings with respect to their main motivating conceptual framework (see Fig. 1), beginning with those close to the ideas of hyperexcitability and impaired excitatory-inhibitory balance in the brain. We will further delve into MEG resting state findings that emphasize a network perspective as a relevant approach to better understand tinnitus, and end with limitations of these works.

In MEG resting state studies, hyperexcitability in tinnitus was initially proposed in the work of Weisz et al. [101] in which reduced alpha and increased delta power was reported for temporal regions in tinnitus patients. This theoretical framework was pursued over the following years, with the work of Schlee et al. [102] being one of the last to almost focus on hyperexcitability exclusively as the underlying cause of tinnitus. Subsequent MEG measurements replicated previous findings of reduced oscillatory alpha power in tinnitus, which was assumed to reflect decreased inhibition—which further leads to an enhanced excitability [101]. Moreover, moment-to-moment variability of power in the low frequency alpha band (8–10 Hz) was significantly decreased in chronic tinnitus, supporting the hyperexcitability hypothesis [102]. Lau et al. [103] on the other hand criticized the hyperexcitability theory, as findings were inconclusive, and some studies did not report alterations in the alpha bands of tinnitus patients [50]. Instead, the authors promoted the thalamocortical dysrhythmia model [34, 104] as well as predictive coding approaches [42, 105]. The authors reported alterations in slow wave and gamma activity to be linked to the perception of tinnitus. Additionally, Lau et al. [103] hypothesized that the heterogeneity of previous results was among other things, due to untargeted confounding variables, such as psychological comorbidities or the circumstances of being in the MEG for the first time. Being novice to MEG measurements did not influence the outcomes; however, differences between the tinnitus and control group vanished in the subgroup without psychological comorbidities [103].

Müller et al. [100] and Hartmann et al. [106] used repetitive transcranial magnetic stimulation (rTMS) to specifically test the theory of excitation-inhibition imbalances with interventional rTMS sessions. Complementing neurostimulation approaches, Hartmann et al. [106] used neurofeedback as an intervention, targeting to increase alpha and reduce delta power in auditory regions. MEG measurements were conducted before and after neurofeedback or rTMS sessions to obtain information about effects on alpha power in the auditory cortex. In terms of neurofeedback, the results of this study align with previous findings, strengthening the positive effects of neurofeedback on altered oscillatory alpha power in the auditory cortex [107, 108]. However, these effects were not found for rTMS, and overall tinnitus was not improved by the stimulation [106]. Müller et al. [100] followed the same approach, and alpha power in the auditory cortex was increased after rTMS interventions. Interestingly, Müller et al. [100] did not solely focus on the auditory cortex but also reported decreased gamma and alpha power in left frontal areas in tinnitus. Hence, they concluded that tinnitus might not solely be generated in the auditory cortex—as previous hyperexcitability approaches assumed—but involves a greater network.

Looking beyond the auditory cortex, Zobay and Adjamian [109] based their MEG resting state study on the thalamocortical dysrhythmia hypothesis [34, 104]. The authors extended their work by hypothesizing enhanced cross-frequency coherence between theta and gamma oscillations, which were assumed to be in line with the thalamocortical dysrhythmia model [34, 35]. However, no significant support for the effect was found in this study, and the authors argued that age and hearing loss could be confounding variables [109]. In a second study, Zobay et al. [110] focused on thalamocortical dysrhythmia as well [34, 104] but extended their theoretical assumptions further to brain network models (global brain model, see [111]). In the auditory cortex, no significant differences in oscillatory power of theta, alpha, and gamma bands were reported between tinnitus individuals and healthy controls. Using connectivity analyses, the tinnitus network approach was further investigated. Evidence for this framework was found with increased functional connectivity in the alpha band within the auditory cortex, as well as increased connectivity in the alpha and beta bands between auditory areas and a global network [110]. This emphasizes the hypothesis that auditory excitation-inhibition imbalances alone are not sufficient for understanding tinnitus but more widespread alterations in various brain regions.

More recent MEG resting state studies were almost exclusively built on alterations in neural networks in tinnitus. Within this framework, Paraskevopoulos et al. [112] focused on directed functional connectivity, building their work on findings of enhanced interactions in tinnitus between cortical networks like limbic, auditory, or attention systems [113–115]. The authors reported enhanced connectivity in tinnitus, indicating increased engagement of attention and emotion networks. These networks are partly located in the dorsal prefrontal cortex—an area that has to be especially stressed since temporal activity was found to be modulated by these prefrontal regions. Therefore, the findings supported the frontal cortex as a relevant aspect of tinnitus. Demopoulos et al. [116] criticized that hearing loss was widely ignored in previous designs, potentially promoting inconsistency among previous findings regarding abnormal neural activity and altered connectivity of functional networks (see, e.g., [14, 106, 110, 117]). Comparing tinnitus patients with normal hearing controls, connectivity was found to be decreased in beta and increased in theta and alpha bands in tinnitus both with and without hearing loss. Increased connectivity was especially found in striatal regions which was interpreted by the authors as support for the striatal gating model [118, 119] which states auditory phantom perceptions as a consequence of dysfunctional connectivity between the striatum and the auditory cortex. As a last study including functional connectivity, Li et al. [120] showed increased connectivity in frontal and temporal areas in tinnitus which was in line with previous findings (e.g., [101, 110]). Alpha band activity was decreased, which indicated reduced inhibition [101]. Delta band power was increased as well, consistent with previous studies indicating interactions between decrease in alpha bands and delta bands [101, 120, 121].

Additionally, in recent research regarding tinnitus in MEG, one work analyzed this topic from a broader biological point of view. Becker et al. [122] focused on peripheral inflammation and its association of oscillatory activity, which was assessed with MEG resting state measurements. In tinnitus, the relevant protein (C-reactive protein; CRP) was found to be negatively correlated with gamma power in the orbitofrontal cortex, meaning that higher CRP levels were associated with decreased activity in the orbitofrontal cortex. Overall, CRP levels were significantly increased in tinnitus compared to the control group. The authors concluded that higher CRP levels and the deactivation of the orbitofrontal cortex are responsible for maintaining tinnitus perception by disinhibiting the auditory cortex. Interestingly, the orbitofrontal cortex was also associated with the tinnitus distress network [17, 123], supporting this framework from a different perspective. Additionally, given the links between chronic inflammation and accelerated biological aging [124], these results strengthen a novel approach towards tinnitus from an aging perspective. Based on epidemiological data, we have recently argued for the importance of identifying putative biological processes that promote accelerated brain aging, leading to an increased proneness to develop tinnitus [60].

Overall, several aforementioned studies based their work on neural network theories [17, 57] and reported findings in line with this framework. Noh et al. [125] aimed to further test the reliability of a wider neural network as an explanation for tinnitus by including interventional dual-site rTMS sessions and conducting MEG resting state measurements before and after the treatments. Next to the auditory cortex, prefrontal regions were targeted as well, and results showed a successful tinnitus suppression after dual-site rTMS sessions, which was enhanced compared to rTMS treatments solely targeting the auditory cortex [126]. Additionally, increased alpha band power was observed after treatments. Overall, the study further supported the theory of a wider network being involved in tinnitus perception by including interventions as a way to test the framework [125].

In conclusion, current MEG resting state studies demonstrate a partly contradictory and inconsistent picture of tinnitus research. Analysis approaches focused both on various oscillatory power bands and different regions to target alterations in tinnitus, but findings were scarcely replicated. Various theoretical foundations and divergent methods between studies further limited the interpretability of findings between studies and confined a deeper understanding of neural alterations in tinnitus.

### Tone Stimulation Paradigms

Aside from resting state measurements, studies have encompassed various designs involving sound stimulation. We will focus mainly on those using simple pure tones. In addition to the findings we obtain from resting state designs, tone stimulations allow researchers to dynamically investigate alterations in the tinnitus brain. Tinnitus perception and neural responses can be analyzed within the influence of external auditory stimulations which allows for a more ubiquitous research on tinnitus [99]. Thus, we will discuss the current existing research using pure tone stimulations in MEG in the following section.

In study designs with pure tone stimulation, researchers commonly investigated evoked and (sometimes) induced responses. As previously mentioned, in auditory research, particularly the auditory steady-state response (ASSR) and the N1/N1m evoked response component are crucial [86, 90]. Similar to the findings in resting state measurements listed earlier, several publications have emphasized hyperexcitability as an underlying cause of tinnitus. According to Sereda et al. [127], increased neural excitability in tinnitus leads to an elevated amplitude of evoked responses. In MEG measurements, different tones corresponding to the tinnitus frequency, a frequency above the audiometric edge in controls, the audiometric edge frequency, and a frequency below the edge were presented. The results showed that N1m amplitudes depended on the tone condition, but this effect did not differ between tinnitus and control groups. Sereda et al. [127] assumed therefore that hearing loss was more related to the pattern differences than tinnitus and did not draw conclusions regarding underlying tinnitus causes. Similarly, Diesch et al. [128] used the ASSR and N1m localized in the primary auditory cortex to investigate the effects of tinnitus and attention. Building on previous studies that emphasized the effect of attention on the ASSR [129, 130], the authors hypothesized that the effects found in tinnitus, which were interpreted as gain control mechanisms [4, 131], might be primarily driven by attention. Stimulation followed the same design as in Sereda et al. [127]. However, the study did not provide evidence for N1m amplitude modulation in tinnitus, and no N1m effect could be attributed to attention. Consequently, the previously reported enhancement of the ASSR in tinnitus [132] could not be solely explained by tinnitus-induced attention shifts [128]. McMahon et al. [133] based their work on the concept of hyperexcitability but additionally proposed map organization processes as an underlying cause of tinnitus. The authors included the 30-week standard Neuromonics Tinnitus Treatment program, which encompasses key aspects such as stimulation of a broad frequency range, music, as well as education and counseling [134]. In addition to the treatment program, tinnitus patients also underwent three MEG recordings while passively listening to different pure tones. Enhanced amplitudes in tinnitus did not change significantly during the treatment. However, shifts in the tonotopic map were found in the tinnitus patients, showing similarities to the control group and supporting the hypothesis of previous map reorganization in tinnitus. Notably, the quantification of the tonotopic map is highly challenging in MEG (see, e.g., [135]). Despite novel approaches to overcome several issues related to the tonotopic map [136–138], interpretations of new findings remain complicated.

Sedley et al. [99] and Adjamian et al. [50] both followed a different approach by basing their studies theoretically on the thalamocortical dysrhythmia hypothesis [34, 35]. In Adjamian et al. [50], the aim was to measure changes in neural activity after modulating the presented stimulus frequency (i.e., sound similar to the tinnitus sensation) by a noise masker. In tinnitus with hearing loss, delta band activity was found to be enhanced in the auditory cortex compared to tinnitus without hearing loss. However, this enhancement diminished when tinnitus was masked with the noise. On the other hand, gamma band activity was not found to be significantly altered in tinnitus. These results suggested that slow wave activity, particularly in the delta band, might play a crucial role in tinnitus perception and partly supported the thalamocortical dysrhythmia. Sedley et al. [99] additionally focused on residual inhibition and excitation. Residual inhibition refers to a decrease in tinnitus intensity after a specific stimulus, which persists even after the sound presentation ends [139]. Residual excitation refers to the opposite phenomenon with increasing tinnitus intensity after stimulus presentation and beyond [99]. During the experiment, both noise maskers and control stimuli were presented. Findings showed that in residual inhibition, tinnitus intensity was positively correlated to gamma band power in the auditory cortex. In residual excitation, the correlation showed an opposite direction. Additionally, oscillatory alterations were also observed beyond the auditory cortex with a higher degree of interindividual variability, indicating relevant neural mechanisms in tinnitus beyond the auditory cortex. Building on these findings, Sekiya et al. [140] focused on aberrant neural activity in tinnitus and broadened population-level frequency tuning in the auditory cortex, which was assumed to reflect inhibitory neural networks [141–144]. Stimuli consisted of the tinnitus frequency of each participant, presented either in isolation or embedded in band-eliminated noises. N1m responses were found to be decreased in the more complex sound condition when presented to the tinnitus ear. Additionally, population-level frequency tuning was broader when sounds were presented to the tinnitus ear, which supported the hypothesis of a maladaptive inhibitory neural network [140].

Overall, these findings partly provided evidence for wider networks as well as map reorganization processes, supporting the assumption that hyperexcitability alone is not sufficient to explain tinnitus. Given the indecisiveness of previous findings, Salvari et al. [145] did not base their work on hyperexcitability but focused on evidence supporting broader networks underlying tinnitus perception [56]. It was further assumed that maladaptive reorganization within these networks consecutively led to tinnitus [146]. Additionally, the authors criticized that most studies analyzing cortical connectivity in tinnitus relied on resting state measurements instead on designs to dynamically detect evoked activity in a larger network. Following the approach of Paraskevopoulos et al. [112], this study used MEG measurements to record evoked responses instead of brain activity during resting state. Stimulation consisted of a control tone of 500 Hz and an individually matched tinnitus frequency. The results demonstrated different processing of the tones in the tinnitus group which was related to reorganizations of processing mechanisms. In comparison to the control tone, the tinnitus frequency elicited a broader network including fronto-temporal, fronto-parietal, and tempo-parietal regions. Interestingly, despite implementing a diverging study design, these findings were in line with previous resting state analyses, further suggesting a broader network to be relevant in tinnitus processes [145]. Moreover, this underlines the aspect of tinnitus patients persistently perceiving their tinnitus sound in resting state measurements.

In another direction of tinnitus research, a focus on the concept of map reorganization led to study designs involving interventions with tailor-made notched music and MEG measurements to examine the effects of the treatments. To implement this, individually chosen music was bandpass filtered to exclude a specific frequency range—most commonly one octave—around the individual tinnitus frequency. MEG measurements were used to record alterations in evoked responses to tone stimulations, including the individual tinnitus frequency and a control tone of 500 Hz. A peculiarity of this study design is that solely tinnitus patients were measured, without the inclusion of healthy control subjects. Moreover, tinnitus patients were mostly selected on narrow criteria, and dropout rates were high. We will describe recent studies that modulated this classical treatment in some respects. In a first attempt, Wunderlich et al. [147] attempted to determine the most sufficient frequency range that must be removed to affect tinnitus. During a 12-week training phase, participants listened daily to music with either a frequency band of one octave, half an octave, or a quarter of an octave removed around the tinnitus frequency. With the MEG measurements, Wunderlich et al. [147] further aimed to investigate potential plasticity effects on tinnitus using the N1m component and the ASSR. While tinnitus related N1m evoked responses were reduced after the training, effects did not show for the ASSR. Moreover, notch width did not have a substantial influence on the outcomes.

Pantev et al. [148] included tinnitus patients who were assigned to either a classical treatment group or a placebo group for which a one-octave range of non-tinnitus related frequencies was removed. All participants were instructed to listen to the music daily for 1 year, and MEG measurements were conducted every 6 months. After 1 year, both tinnitus loudness and related auditory activity were significantly reduced in the treatment group compared to the placebo group. The improvement was attributed to the effect of the music treatment which led to a normalization of the inhibition-excitation balance. Additionally, the results indicated a long-term neuroplastic effect that counteracted maladaptive cortical reorganization. Pape et al. [149] built their work upon these findings of Pantev et al. [148]. They divided the tinnitus patients into two groups. The unimodal group was instructed to pay attention to the music and detect auditory variations between repetitions of songs. The multimodal group, on the other hand, was instructed to play melodies on a tablet while listening to music songs—hence attention was divided between auditory, visual, and somatosensory modalities. During a 2-month period, participants had daily training sessions specific to their assigned group task, and three MEG measurements were conducted (before, during, and after the training phase). The results showed that in the unimodal group, cortical activity corresponding to the tinnitus frequency was decreased, while such a change was not observed in the multimodal group. Interestingly, changes were not only reported in the auditory cortex but also in posterior parietal regions—which are part of a supposed tinnitus network [17]. This effect is in line with previous studies [148, 150], showing reduced tinnitus perception and reduced activity in the auditory regions corresponding to the tinnitus frequency. Overall, these findings indicated that maladaptive reorganizations are relevant in tinnitus and can be reversed [149].

Stein et al. [151] based their work on a shorter period of time with training sessions lasting 3 h on three consecutive days. The music was notch-filtered with a bandwidth of 0.5 octaves around the tinnitus frequency. After the training, inhibition-induced plasticity was observed not only in the auditory cortex but also in a wider distributed network of temporal, frontal, and parietal regions. These findings supported the theory of a wider network being involved in tinnitus [21, 54]. Interestingly, neural reorganization appeared to occur rapidly after only short periods of music training [151]. In a second work, Stein et al. [152] followed the same approach. In addition to a participant group listening to music with a notch filter of 0.5 octaves bandwidth around the tinnitus frequency, a second group of participants listened to music with an additional 20 dB amplification of frequency bandwidths of ^3^/_8_ octaves on each side of the notch (increased spectral energy contrasts; ISEC). Previous studies had shown that this procedure induced inhibition of neurons coding the frequency at the center of the notch (i.e., the tinnitus frequency) [153]. For the group with classical tailor-made notched music training, tinnitus loudness and related neural activity were reduced—not only in the auditory cortex but also in temporal, parietal, and frontal regions of the tinnitus network [54, 101]. These findings were in line with the previous results [151]. In the second group including ISEC procedures, additional clusters of reduced neural activity were found in temporal and prefrontal regions. This further strengthened the claim of a wider tinnitus network. Additionally, the study showed that increased spectral energy contrast led to more pronounced plasticity compared to classical procedures [152].

To further evaluate the map reorganization theory in tinnitus, Li et al. [154] included interventional rTMS sessions to analyze the functional organization of the auditory pathway. This study aimed to build up on their previous work, which had indicated lesion induced changes in neuroplasticity and subsequent reorganization processes [155, 156]. The authors focused on the steady-state auditory evoked fields, which were shown to be increased in tinnitus [157]. One month after rTMS treatments, steady-state auditory evoked fields were decreased in tinnitus patients which demonstrated the first findings of long-lasting rTMS effects on tinnitus and supported the map reorganization theory in tinnitus [154].

## Shortcomings and Problems of the Field

This overview of findings in MEG regarding tinnitus displays a great variety of theories and approaches to target tinnitus. On the one hand, resting state measurements are a powerful method to target neural networks and impairments related to altered excitatory and inhibitory processes in the brain. On the other hand, a proportion of research in this field focuses on tone stimulations to investigate primarily reorganization processes in tinnitus. As the description of recent work demonstrated, different theories were included to approach tinnitus, and findings were highly inconsistent. The current imprecision in clearly mapping theoretical foundations onto neural processes repeatedly leads to inconsistent findings which limits the advances in tinnitus research. Additionally, as mentioned earlier, neuro-developmental aspects and the influence of time are widely ignored (axis C in Fig. 1). Regarding neurodevelopmental aspects, the underlying mechanisms are further unknown, such as potential pathological processes or cognitive changes that lead to a higher reliance on “priors” in line with the Bayesian inference framework. A more comprehensive approach to tinnitus would allow us to derive a more comprehensive and reliable view on this complex phenomenon.

As a second caveat, tinnitus research in humans often fails to link their concepts and results to previously established animal models. There exists a long line of tinnitus research on various animals that could provide a better understanding and interpretation of findings in human research. For instance, inconclusive findings in humans regarding the hyperexcitability theory are also well reported in animal models. Research in mice, rats, and guinea pigs demonstrated that enhanced excitability could not be found in every animal, restricting the assumption of hyperexcitability as the only underlying mechanism for tinnitus (see, e.g., [51–53]). Next to extensive research on rodents or cats [158], research on primates additionally allows to investigate the role of the frontal cortex in tinnitus [159]. Animal models comprise concepts of cellular, molecular, or pathophysiological features that cannot be derived from human subjects [158] since they allow for invasive procedures. Additionally, animal studies further allow to control over the assignment of individual animals to experimental groups and standardized noise exposures. However, in comparison to human subjects, animals lack the advantage of verbal communication and face reliability and validity issues [158, 160]. Moreover, in terms of tinnitus distress, we are not able to derive the extent of tinnitus severity from animals which limits the interpretability of animal models. Nevertheless, concerted efforts to integrate animal and human models are needed to deepen our understanding of tinnitus generation of humans [15, 161].

In conclusion, these existing caveats constrain current tinnitus research and need to be addressed and overcome.

## Future Directions and Guidelines

Although tinnitus has been extensively investigated using MEG in the last years, the field has largely stagnated and requires novel impulses and innovations. Based on the recent findings that mainly focused on a specific aspect of tinnitus models, we suggest a more integrated approach to tinnitus that incorporates the aspect of time (see axis C and Fig. 1). Age has previously been associated with the prevalence of tinnitus ([2, 162, 163] for a negative finding, see [164]), but it is not embedded as an independent factor in current tinnitus models. Despite the statistical correlation, the aging process is typically not considered as a risk factor itself but rather attributed to age-related hearing loss [165]. However, we propose that aging processes might act as risk factors in their own right. Al-Swiahb and Park [166] demonstrated that the greatest increase in tinnitus onset occurs around middle age (< 60 years), preceding the peak increase in the onset of hearing loss. Moreover, we recently demonstrated in two different samples that tinnitus risk is increased in older adults—independently of hearing loss [60]. However, chronological age does not reflect latent biological aging processes that potentially influence tinnitus development or increase vulnerability. We propose a conceptual integration from a brain aging viewpoint to link hearing loss and tinnitus to biological aging processes. This novel approach allows us to further advance in the field and deepen our understanding of biological processes in the brain that are related to tinnitus.

As an addition to this new theoretical approach, several conceptual and methodological options can be addressed to improve future tinnitus research in MEG. Novel paradigms are needed to comprehensively investigate tinnitus and determine relevant processes and alterations in the brain. We suggest not only investigating brain reactions to stimulations or interventions but also focusing on prediction tendencies as a predisposition to tinnitus. In line with the Bayesian inference framework [42, 45], the brain builds predictions that are compared to incoming sensory input and used to update internal models. In tinnitus, increased spontaneous activity along the auditory pathway leads to a change in these predictions. The default prior of silence is altered to the prediction of a sound, which is perceived as tinnitus [42]. Recent work supported this concept of altered predictions in hallucinations and tinnitus, but the underlying mechanisms remain unclear [48, 49, 167]. In this vein, we recently observed marked differences to the visual modality [168], which does not exhibit the same “passive” anticipatory predictions as observed in audition [169]. We suggest future research to emphasize this line of research for a deeper understanding of underlying mechanisms and individual differences that explain the vulnerability and occurrence of tinnitus in certain individuals.

From a methodological perspective, we suggest integrating more machine learning techniques, which are still sparse in current analysis approaches (see, e.g., [170]). Additionally, source analyses depict a powerful technique beyond analyses on sensor level to extract more precise localizations of relevant neural activity. However, many studies focus solely on analyses at the sensor level and do not take a step further to estimate the neural source of altered activity in tinnitus. Moreover, another caveat of current research approaches is the lack of differentiation between periodic and aperiodic components within the MEG signals, with a widespread tendency to ignore aperiodic parts of the data [171]. Consequently, several possibilities to target neural activity in tinnitus are not implemented and utilized yet and should be considered in future study designs as well.

Another important development will be the usage of optically pumped magnetometers (OPMs, [172]). This novel technology marks a significant advancement in human neuroscience, providing a novel means of measuring the magnetic fields generated by brain activity. The portable and less “invasive” nature of OPMs offers distinct advantages in comparison to traditional MEG system, first of all ensuring higher ecological validity. OPMs enable researchers to examine how diseases’ (e.g., tinnitus) symptoms manifest and fluctuate in daily life scenarios, an aspect critical to developing effective treatment strategies.

For a deeper understanding of the underlying tinnitus mechanisms, it is further not sufficient to focus entirely on individuals with chronic tinnitus. A differentiation has to be made between individuals that suffer from acute tinnitus that disappears after several weeks and individuals that develop chronic tinnitus. At this point we do not have sufficient insights in how acute tinnitus patients differ from individuals with chronic tinnitus. Additionally, research lacks in investigations of individuals undergoing successful tinnitus treatment. Future research should target these two groups more specifically to gain knowledge about underlying mechanisms and brain correlates that are responsible for the development of chronic tinnitus.

In conclusion, extensive work has already been accomplished in the field of tinnitus, and new findings have been revealed over the last years using MEG as a powerful research tool. However, more work needs to be done on a more comprehensive theoretical foundation, and new conceptual, methodological, and technical approaches should be incorporated to gain new insights into the neural mechanisms of tinnitus.

### Supplementary Information

Below is the link to the electronic supplementary material.

Supplementary file1 (DOCX 10 KB)
