# Supplementary material for: Eavesdropping on Tinnitus Using MEG: Lessons Learned and Future Perspectives
Source: J Assoc Res Otolaryngol. 2023 Nov 28;24(6):531–47. doi: 10.1007/s10162-023-00916-z (PMC10752863; doi:10.1007/s10162-023-00916-z)
Supplement: Supplementary file 1 — Supplementary file1 (DOCX 10 KB) [file 10162_2023_916_MOESM1_ESM.docx]

**Supplementary**

***Table 1:*** *Overview of published tinnitus research in MEG over the past 10 years (resting state measurements).*

| Authors | Title | Theoretical foundation | Key findings |
| --- | --- | --- | --- |
| Müller et al., 2013 | rTMS Induced Tinnitus Relief Is Related to an Increase in Auditory Cortical Alpha Activity | Inhibition-excitation imbalance | - increased alpha power in the auditory cortex after rTMS interventions  - decreased gamma and alpha power in left frontal areas in tinnitus |
| Hartmann et al., 2014 | The Effects of Neurofeedback on Oscillatory Processes Related to Tinnitus | Inhibition-excitation imbalance | - neurofeedback interventions led to an increase of alpha power in tinnitus  - effect could not be reported for rTMS and overall tinnitus was not improved |
| Schlee et al., 2014 | Reduced Variability of Auditory Alpha Activity in Chronic Tinnitus | Hyperexcitability | - reduced alpha power in tinnitus  - moment-to-moment variability of power in the low frequency alpha band (8-10 Hz) was decreased |
| Zobay et al., 2015 | Source Space Estimation of Oscillatory Power and Brain Connectivity in Tinnitus | Thalamocortical dysrhythmia  Neural networks | - no alterations in the auditory cortex in theta, alpha and gamma power in tinnitus  -increased functional connectivity in the alpha band within the auditory cortex  - increased connectivity in alpha and beta between auditory areas and a global network |
| Zobay and Adjamian, 2015 | Source-Space Cross-Frequency Amplitude-Amplitude Coupling in Tinnitus | Thalamocortical dysrhythmia | - no significant coherence between theta and gamma oscillations  - age and hearing loss were assumed to be confounding variables |
| Lau et al., 2018 | Targeting Heterogeneous Findings in Neuronal Oscillations in Tinnitus: Analyzing MEG Novices and Mental Health Comorbidities | Thalamocortical dysrhythmia  Predictive coding framework | - increased slow wave activity in tinnitus  - psychological comorbidities influenced group differences  - being novice to MEG measurements did not confound the results |
| Paraskevopoulos et al., 2019 | Maladaptive alterations of resting state cortical network in Tinnitus: A directed functional connectivity analysis of a larger MEG data set | Neural networks | - enhanced connectivity in tinnitus interpreted as increased engagement of attention and emotion networks |
| Noh et al., 2020 | Treatment Outcome of Auditory and Frontal Dual-Site rTMS in Tinnitus Patients and Changes in Magnetoencephalographic Functional Connectivity after rTMS: Double-Blind Randomized Controlled Trial | Neural networks | - successful tinnitus suppression after dual-site rTMS sessions (auditory and prefrontal regions)  - dual-site rTMS showed stronger effects compared to rTMS treatments solely on the auditory cortex  - increased alpha band power after treatments |
| Demopoulos et al., 2020 | Global resting-state functional connectivity of neural oscillations in tinnitus with and without hearing loss | Neural networks  Striatal gating model | - comparing tinnitus patients with normal hearing controls, connectivity was decreased in beta and increased in theta and alpha bands in tinnitus |
| Becker et al., 2022 | Higher Peripheral Inflammation Is Associated With Lower Orbitofrontal Gamma Power in Chronic Tinnitus | - | - tinnitus relevant protein (C-reactive protein; CRP) was negatively correlated with gamma power in the orbitofrontal cortex (i.e., decreased activity) - CRP levels increased in tinnitus |
| Li et al., 2022 | Pros and cons in tinnitus brain: Enhancement of global connectivity for alpha and delta waves | Hyperexcitability  Neural networks | - increased connectivity in frontal and temporal areas in tinnitus  - decreased alpha band activity and increased delta power indicated enhanced inhibition |

***Table 2:*** *Overview of published tinnitus research in MEG over the past 10 years (tone stimulation paradigm).*

| Authors | Title | Theoretical foundation | Key findings |
| --- | --- | --- | --- |
| Diesch et al., 2012 | Is the effect of tinnitus on auditory steady-state response amplitude mediated by attention? | Hyperexcitability | - no evidence for N1m amplitude modulation in tinnitus  - no N1m effect of attention  - enhancement of the ASSR in tinnitus (Diesch et al., 2010) are not due to tinnitus induced attention shifts |
| Pantev et al., 2012 | Tinnitus: the dark side of the auditory cortex plasticity | Map reorganization | - after one year of music intervention, tinnitus loudness was reduced  - related auditory activity was reduced as well  - the results indicated a long-term neuroplastic effect which counteracts cortical reorganization |
| Sedley et al., 2012 | Single-subject oscillatory gamma responses in tinnitus | Thalamocortical dysrhythmia | - relevance of gamma-band oscillations in inhibiting abnormal cortical activity and attenuating perception in tinnitus  - oscillatory alterations beyond the auditory cortex with high interindividual variability |
| Adjamian et al., 2012 | Neuromagnetic Indicators of Tinnitus and Tinnitus Masking in Patients with and without Hearing Loss | Thalamocortical dysrhythmia | - enhanced delta band activity in tinnitus in the auditory cortex  - diminished delta band activity in a masking condition - gamma activity was not linked to tinnitus while masking |
| Sereda et al., 2013 | Auditory evoked magnetic fields in individuals with tinnitus | Hyperexcitability | - N1m amplitudes depended on different tone conditions  - hearing loss was more related to the differences than tinnitus |
| Pape et al., 2014 | Playing and Listening to Tailor-Made Notched Music: Cortical Plasticity Induced by Unimodal and Multimodal Training in Tinnitus Patients | Map reorganization | - cortical activity corresponding to the tinnitus frequency was decreased in a patient group that paid attention to music deviations  - effect not found in a multimodal group with various tasks  - increased activity in posterior parietal regions additionally to the auditory cortex were also found |
| Wang et al., 2015 | Extractions of steady-state auditory evoked fields in normal subjects and tinnitus patients using complementary ensemble empirical mode decomposition | Map reorganization | - steady-state auditory evoked fields were increased in tinnitus |
| Wunderlich et al., 2015 | Impact of Spectral Notch Width on Neurophysiological Plasticity and Clinical Effectiveness of the Tailor-Made Notched Music Training | Map reorganization | - tinnitus related N1m evoked responses were reduced after training with tailor-made notched music  - effects did not show for the ASSR  - notch width did not have an influence |
| Stein et al., 2015a | Inhibition-induced plasticity in tinnitus patients after repetitive exposure to tailor-made notched music | Map reorganization | - inhibition-induced plasticity after training was found in the auditory cortex and in a wider distributed network of temporal, frontal and parietal regions  - neural reorganization appeared fast after short periods of music training |
| Stein et al., 2015b | Enhancing inhibition-induced plasticity in tinnitus–spectral energy contrasts in tailor-made notched music matter | Map reorganization | - classical tailor-made notched music training reduced tinnitus loudness and related neural activity  - effects in the auditory cortex and in temporal, parietal and frontal regions of the tinnitus network  - in a group including increased spectral energy contrasts procedures, neural activity was additionally reduced in temporal and prefrontal regions |
| McMohan et al., 2015 | Cortical Reorganisation during a 30-Week Tinnitus Treatment Program | Hyperexcitability  Map reorganization | - no amplitude changes in tinnitus during the Neuromonics Tinnitus Treatment program  - shifts in the tonotopic map in tinnitus patients showed similarities to source locations of the control group |
| Sekiya et al., 2017 | Broadened population-level frequency tuning in the auditory cortex of tinnitus patients | Neural networks | - decreased N1m responses in a more complex sound condition (tinnitus frequency embedded in band-eliminated noises)  - frequency tuning was broader when sounds were presented to the tinnitus ear |
| Li et al., 2019 | Steady-state auditory evoked fields reflect long-term effects of repetitive transcranial magnetic stimulation in tinnitus | Map reorganization | - steady-state auditory evoked fields were decreased in tinnitus patients one month after rTMS treatments  - first findings of long-lasting rTMS effects on tinnitus |
| Salvari et al., 2023 | Tinnitus-frequency specific activity and connectivity: A MEG study | Neural networks  Map reorganization | - reorganizations of processing mechanisms in tinnitus led to divergent processing of tones  - presentation of the tinnitus frequency elicited a broader network of fronto-temporal, fronto-parietal and tempo-parietal regions |
